# Perspectives: This house believes that a natural facet of nursing work is that it depletes nurses’ wellbeing

**DOI:** 10.1177/17449871211014021

**Published:** 2021-06-05

**Authors:** Pam Smith, Gearóid K Brennan, Daniela Mansilla Castillo, Radha Adhikari

**Affiliations:** Professorial Fellow, Department of Nursing Studies, University of Edinburgh, UK; Clinical Teaching Fellow, Department of Nursing Studies, University of Edinburgh, UK; Department of Psychological Medicine, RIE, NHS Lothian, UK; PhD Student, Department of Nursing Studies, University of Edinburgh, UK; Lecturer, School of Health in Life Sciences, University of the West of Scotland, UK

## A note from Pam Smith

When JRN editors Andree and Ann approached me to propose the motion how nurses’ work depletes their wellbeing, I was excited by their invitation to convene a team to propose and debate the motion. I was fortunate to be able to call on such esteemed colleagues from a range of specialist and international perspectives to prepare the debate. Daniela Castillo reflects that during our preparation, the more we shared ideas and learning the more we agreed that nursing work is emotional work the world over. Nurses’ emotional labour has clearly been exacerbated during the coronavirus 2019 (COVID-19) pandemic. With hospitals overcrowded and supplies running short, the risk of contracting the disease we were fighting was very real. In some places, nurses were being recognised by the population, clapping us as heroes, yet in others were getting spat at or evicted for being a front-line worker and a risk to the general public. However, our aim in the debate was to show that the depletion of nurses’ wellbeing is a perennial problem long before COVID-19 hit the world.

Our motion was carried with 65% of the 136 international participants voting to support our case ‘that a natural facet of nursing work is that it depletes nurses’ wellbeing’. Following the debate, even the inspiring Jos de Blok and his Netherlands Neighbourhood Nursing Team could only convince a further 10% of the audience to oppose our motion. This is a sobering result and calls for action to recognise and support nurses’ vital contribution to people’s health at the same time as protecting their wellbeing. In writing this Perspectives article, we have revisited our case for the motion, which inspired four themes to frame a call for action. These are: the nature of nursing work; invisibility of nursing work; internal negotiation; and nurses’ experiences are global and collective. These themes recognise nurses’ vital contribution to healthcare and the part played by emotional labour in both protecting and depleting their wellbeing.

## Nursing work

Our case for the motion started with me, Pam Smith. I wanted to put nursing work in a global context and so I began with a few facts and some questions about the nature of nursing work and its impact on wellbeing. Globally nursing accounts for 59% of all healthcare professionals and 90% are women. Nurses are seen as central to achieving universal health coverage, meeting the Sustainable Development Goals (SDGs) and to have a ‘triple impact’ towards achieving greater gender equality, better health and stronger economies (World Health Organization, 2016). Huge expectations are put on nurses despite a global shortage of about 6 million.

Yet what is the nature of nursing work? Nearly 50 years ago nursing and midwifery were declared the major caring professions (Department of Health and Social Security, 1972). Much of nurses’ work remains unnoticed, uncounted and undervalued (Ruchti, 2012). Nurses’ UK pay actually dropped 20% over 10 years. But nursing work only remains unnoticed until things go ‘wrong’; then nurses become scapegoats for national scandals.

It is not surprising therefore that nurses’ mental health is suffering and ‘at breaking point’, a headline which appeared long before the COVID-19 pandemic.

So, what is ‘natural’ about the caring work of nurses? The assumption surely arises because 90% of nurses are women, so nursing work is taken for granted as just women’s work. My own research showed otherwise. When I spoke to student nurses they described the sheer hard work required to maintain a smile or stay calm to care for patients. Nurse leaders were vital because they set the emotional tone. As one student put it ‘They are critical because of their influence on the ward team and how the students work, the way they feel, their morale’. When students knew their leaders cared, they felt ‘a bit more at ease’. When they didn’t, they felt ‘fear’ which blocked their learning. Mutual respect was the best and supported them to do the ‘little things’ to care for patients to help their ‘emotional side’ (Smith, 1992, 2012).

Sociologists give a language to connect emotions, care and women’s work. Graham sums it up: ‘When we talk about caring for someone we are talking about our emotions’ (Graham, 1983). The theory of ‘emotional labour’ of Hochschild (1983, 2003) describes the effort required to undertake the often invisible ‘people work’ of service sector workers. Put simply emotional labour requires ‘induction or suppression of feeling to present an outward appearance of calm to produce in others a sense of being cared for in a convivial, safe place’ (Hochschild, 1983, 2003: p. 7). Emotional labour is exchanged for a wage and has commercial value.

Why does the nature of nursing work deplete nurses’ wellbeing? This happens when their ‘taken for granted’ emotional labour becomes a burden not only to themselves but also to patients, colleagues and families. The commercial value of their emotional labour may also deplete their wellbeing as suggested in a study of Canadian health services. The pressure to meet financial targets limited nurses’ decision making and forced them to go against their expert judgement resulting in conflict and dissonance, stress and burnout (Rankin and Campbell, 2006).

As so many COVID-19 stories reveal, nurses are a constant on the frontline of global healthcare, working with limited resources to face not only the pandemic but also wars, political conflicts and emergencies, while giving extraordinary care to people at the extremes of living and dying. The COVID-19 pandemic accentuates these extremes to deplete nurses’ wellbeing, by increasing the intensity of their work to risk emotional detachment, burnout and post-traumatic stress disorder (PTSD).

Yet despite this, patients recovering from COVID-19 describe the extraordinary ways nurses cared for them. British author Michael Rosen talks about the ‘letters of hope and support’ written by nurses, which helped him survive.

The pandemic reveals just what nurses do but also exposes the inequalities of class, gender, race and ethnicity and the vulnerabilities of migrant and refugee communities. We must urge governments to recognise nurses’ emotional labour, how it is integral to the caring economy and vital to gender equality, sustainability and wellbeing.

Gearóid Brennan takes up our case in the second theme.

## Nursing work and invisibility

In order to discuss how nurses’ work depletes wellbeing, we need to understand what constitutes ‘nursing work’. The majority of nursing work is unseen because it is what Allen (2014) coins ‘organising work’. This involves information seeking, making sense of complexity, communication and the use of information technologies. It is nurses’ interaction and negotiation with multiple systems at the same time in order to keep patients progressing within our healthcare system. Nurses’ work is not confined to the bedside in medical wards but takes place in a multitude of settings including general practitioner (GP) practices, schools, sports events, people’s own homes and in universities, as nurse educators.

Therefore, the nature of the work will depend on the setting and role. In my own work, this ranges from undertaking biopsychosocial psychiatric assessment in a busy accident and emergency department to delivering learning to undergraduate students in the now ‘virtual’ classroom. As well as the actual face-to-face interaction in both of these scenarios, there are a lot of things that are not so visible. This includes making autonomous decisions about what mental health interventions someone may require, assessing and managing risk as well as liaising with other parts of the health and social care system; family members, GPs, support workers, voluntary organisations, bed managers, community mental health teams, my own colleagues. In the university, there are times when there is equally as much ‘invisible’ work that takes place outside the classroom but which is essential to the development of nursing students who are the future workforce.

All of these things are nursing work and the crux of the issue is that all roles have two things in common; relationships and structures. As someone who delivers interpersonal psychotherapy, other people are ‘the hell in our lives’ but they are also the source of much joy. We experience this to some degree every day. Why then would it be any different for nursing, a profession that prides itself on interpersonal relationship building? Regardless of the type of ‘nursing work,’ nurses work with people, listen to their stories and try and relate. It is because of this essential element, this interpersonal nature of the work, that in my opinion, risks nurses’ wellbeing.

This is because nurses often end up listening to people when they are demonstrating heightened levels of expressed emotion; patients, students, research participants, colleagues! It is this interpersonal component that brings about emotional labour. This too is often invisible. Frequently some of the distress which people express is down to systems and structures far beyond nurses’ scope of influence, yet they remain the face of these systems; all the responsibility with none of the power.

This emotional labour is magnified because so many of the systems don’t function very well. And herein lies the distress for nurses; they see that there is a person at the centre of all this – a patient or a student. And often what is required of nurses is to engage in ‘organising work’ for the sake of the individual and come up against barrier after barrier. This frustration is what contributes to depleting wellbeing. And what is worse is that this is often invisible and not recognised for what it is: essential to keeping the system going. Allen (2014) herself states that the better this is done the less visible it is to those who benefit from it. And it is this constant invisibility of nurses’ interaction with the interpersonal (agency) and systems (structures) that mean our wellbeing is constantly being depleted.

Daniela Castillo presents further evidence for our case in the third theme.

## Internal negotiation

The topic under debate is pivotal to discuss if a decrease in nurses’ wellbeing can directly affect the level of care that they are able to provide and, therefore, affect patients’ outcomes and the capacity of entire institutions to care. Interestingly, as a nurse from Chile, I am amazed that our experiences from such varied and distant contexts converged on so many points. In any age or place, nurses have always had a need for internal negotiation between structure and agency even though this remains invisible to health services. Dissonance of beliefs, values, emotions and the demands of the organisation to meet the needs of patients and families, maintained within a context and relative to others, are part of the hidden daily struggles, outwardly displaying our professional knowledge in practice.

Emotional wellbeing is not seen as important in the intricate architecture of care. In this way, we work as a divided, disconnected individual, with the false idea of dualism between head and heart. But even more problematic is the belief that providing care is an individual responsibility. The ideas of Madeleine Bunting (2020), the author of *Labours of Love: The Crisis of Care*, became something that helped me understand the cost-neutrality of the nurses’ emotional labour in the provision of care. This was something that I tried to exemplify in the debate through real experiences. Feelings of isolation in the workplace and being split between thoughts and emotions hinders agency, depletes wellbeing and is commonplace in the nursing profession.

During the debate, my aim was to connect the audience with the emotional tone of clinical nursing work and the everyday contradictions that are negotiated through my own story. Joy and burden are two sides of the same coin. On the one hand, being a nurse has contributed to giving meaning and purpose to my life, having the privilege of transforming and positively impacting on the experience of the patients I have cared for. On the other hand, I have been overwhelmed on a professional and personal level. In my experience, the lack of tools at the individual, relational and organisational level to deal with emotions therapeutically when witnessing the suffering of patients and relatives added to the demands of the organisation, and deterioration in the quality of care. I have witnessed how this emotional burden has triggered physical ailments and pervaded my personal and family relationships.

External and internal factors are juxtaposed in these negotiations. Nurses make decisions often in hostile work environments, with scarce resources, low autonomy, and little or no training in managing emotions to deal with the emotional labour that nursing work implies. The debate from my perspective is not to assume blame on either the organisational system or the way nurses handle nursing work to be responsible for the depletion of their wellbeing. This way of thinking clings to the prevailing dichotomy. Rather, we need to move from an individual approach to a broader systems-level approach to providing care; and to make the many obstacles visible during these negotiations.

Radha Adhikari now considers our case for the fourth and final theme.

## Nurses’ experiences are global and collective

As the motion suggests, nursing, the very profession that supports people to regain health and promote their wellbeing, depletes its workforce’s wellbeing.

Globally, nurses face various occupational health risks, among them: requiring them to work under an increasingly pressurised modern healthcare environment and usually long and unsocial hours. Nurses are not only exposed to healthcare-related infections, but it is also the case that their provision of person-centred and compassionate care takes its toll on their own physical and emotional wellbeing.

The build-up of stress can be a slow process, and may not always be visible to others. Nurses manage their negative emotions by supressing them, as they know they need to appear confident, competent and emotionally strong, so as, by instilling confidence, they can promote positive health outcomes for their patients. The COVID-19 and past pandemics have demonstrated, only too clearly, the extent of nurses’ daily exposure to life-threatening infections and to the extremes of stress.

The scope of nursing practice, as regards nurses’ roles and public expectations, is constantly changing, which increases the pressure on nurses to remain efficient and up-to-date in all areas. Nurses need to learn to manage new healthcare challenges, new technology and ever-changing healthcare guidelines. Research evidence suggests that, as a result of working in extremely pressurised environments, the rate of burnout in the frontline nursing workforce is high. For example, in China as many as 64% of nurses experienced burnout (Qing-Qing et al., 2019). In the UK, NHS staff regularly report job burnout (Campbell, 2019). The COVID-19 crisis and similar pandemics regularly compound and magnify this situation globally (Magill et al., 2020). This phenomenon is widespread and global, and demands an increase in the workforce.

Currently, the shortfall in the nursing workforce is a national and indeed global issue. There is a mismatch between demand and supply at all levels, and most countries in the world are facing these critical shortages. There are a number of reasons for countries lacking sufficient numbers of staff. Among them are: the gendered nature of nursing work; unsocial hours; low pay; and poor or unsafe working conditions. Unfortunately, it was once regarded as a profession for life, but this is no longer the case. Due to health service reforms and cost cutting mechanisms, many nurses are leaving the NHS in the UK or remaining inactive, and many others are in a ‘should I stay or should I go’ dilemma (McGrath, 2006).

A recent Royal College of Nursing (RCN) UK survey suggests that as many as 36% of nurses were considering leaving the profession, pay being the main reason behind their decision (Royal College of Nursing, 2020). Frontline nurses feel forgotten by the government, like one of our colleagues in London, who not only felt ‘undervalued’ but as if they have been ‘slapped in the face by the government’. Witness the recent pay review for public service workers, in which nurses received no recognition or pay rise. The government applause now appears a patronising and hollow gesture. The recent 1% pay increase is further evidence of this. The paltry award was vigorously defended by Conservative Lord Bethell’s claim that: ‘nurses are well-paid’ for ‘a secure job and have other benefits’ (Mitchell, 2021).

To compound this issue, student nurses’ drop-out rate in the UK has been worryingly high, as high as 24% of the total intake in 2017 and 2018 (Perry, 2019). In affluent countries this profession’s attractiveness is declining. However, February 2021 figures show a surprising rise in student nurse applications, attributed to the pandemic’s exposure of ‘the poignant and essential nature of nursing work’ (Open Access Government, 2021). This rise in interest is unlikely to compensate for the vast numbers retiring and quitting the profession due to work-related stress and poor working conditions.

Recent popular representations of nurses have been as ‘NHS Heroes’, or ‘Corona Virus Heroes’ (McKoy, 2020), and as life-saving angels. The key point, which should never be forgotten is, of course, that nurses are not invincible. They cannot just be relegated to the above categories, and so defined, for, more importantly, they must be seen as human beings. Like anyone else, nurses deal with a variety of personal and professional emotional issues, nurses have to manage work and family expectations and deal with work-related pressures from management. All of which impacts on their personal lives. So, the work nurses do definitely challenges their health and overall wellbeing. Health systems across the world should recognise this and work towards supporting their wellbeing.

## Conclusions

Gearoid reveals the complexity and interpersonal nature of all nursing work and the importance of systems to support nurses’ mental health. Yet the majority of nurses do not have access to relevant supports such as clinical supervision, which is still not mandatory. Daniela emphasises the joy and burden of caring and the risk of dissonance; Radha describes the personal, physical and emotional costs of caring which depletes nurses’ wellbeing. She reminds us that nurses are only human, but therein lies our humanity and commitment to care. Our motion is pivotal because any decrease of nurses’ wellbeing can directly affect levels of care they are able to provide both to themselves, each other, patients, families and the capacity of institutions and indeed nations, to cope and care.

We conclude with the inspiring words of Eduardo Galeano, a Uruguayan author suggested by Daniela to reflect on the emotional labour of care:

Me gusta la gente sentipensante, que no separa la razón del corazón. Que siente y piensa a la vez. Sin divorciar la cabeza del cuerpo, ni la emoción de la razón.

I like feeling-thinking people, who do not separate the reason from the heart. Who feel and think at the same time, without divorcing the head from the body, and neither the emotion from the reason.

## Key points for policy, practice and/or research

- Globally Nursing accounts for 59% of all healthcare professionals and 90% are women.
- Nursing is complex caring work which requires internal negotiation.
- Nurses' experiences are global and collective and involve personal, physical and emotional costs.
- Systems need to be in place to support nurses' mental and physical health and promote their wellbeing.
- The emotional labour of nursing provides a lens to recognise the joy and burden of caring and the need for adequate financial reward.
